# Understanding PI3K/Akt/mTOR signaling in squamous cell carcinoma: mutated PIK3CA as an example

**DOI:** 10.1186/s43556-024-00176-0

**Published:** 2024-04-15

**Authors:** Shutao Zheng, Shuo He, Yan Liang, Yiyi Tan, Qing Liu, Tao Liu, Xiaomei Lu

**Affiliations:** 1 State Key Laboratory of Pathogenesis, Prevention and Treatment of High Incidence Diseases in Central Asia, Clinical Medical Research Institute, The First Affiliated Hospital of Xinjiang Medical University, Urumqi, 830054 Xinjiang People’s Republic of China; 2 Department of Clinical Laboratory, First Affiliated Hospital of Xinjiang Medical University, Urumqi, 830054 Xinjiang People’s Republic of China

**Keywords:** PI3K, PIK3CA, Mutation, Squamous cell carcinoma, Resistance

## Abstract

Compared with those in adenocarcinoma, PIK3CA mutations are more common in squamous cell carcinoma (SCC), which arises from stratified squamous epithelia that are usually exposed to adverse environmental factors. Although hotspot mutations in exons 9 and 20 of PIK3CA, including E542K, E545K, H1047L and H1047R, are frequently encountered in the clinic, their clinicopathological meaning remains to be determined in the context of SCC. Considering that few reviews on PIK3CA mutations in SCC are available in the literature, we undertook this review to shed light on the clinical significance of PIK3CA mutations, mainly regarding the implications and ramifications of PIK3CA mutations in malignant cell behavior, prognosis, relapse or recurrence and chemo- or radioresistance of SCC. It should be noted that only those studies regarding SCC in which PIK3CA was mutated were cherry-picked, which fell within the scope of this review. However, the role of mutated PIK3CA in adenocarcinoma has not been discussed. In addition, mutations occurring in other main members of the PI3K-AKT-mTOR signaling pathway other than PIK3CA were also excluded.

## Introduction

**Fig. 1:**
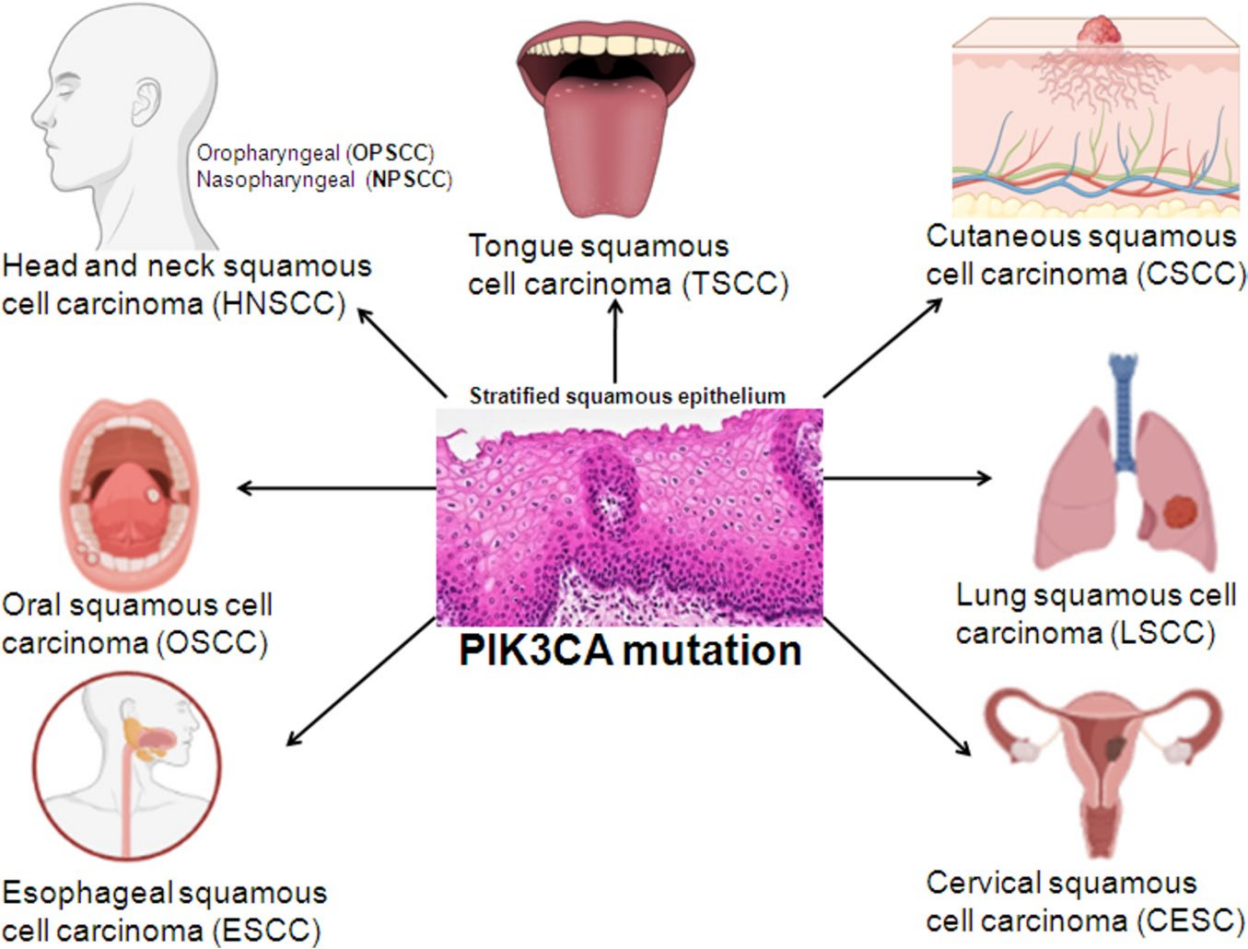
PIK3CA mutation in squamous cell carcinoma (SCC) originating from stratified squamous epithelium of head and neck, oral, esophageal, tongue, skin, lung and cervical tissues. The icons representing the human head and neck, mouth, esophagus, tongue, skin, lung and cervix were credited to the free-trial version of the online drawing platform (https://www.biorender.com/)

Commonly observed in cancer, PIK3CA mutation is second only to TP53 mutation. Although many articles have even reviewed papers investigating or summarizing the biological and clinical roles of mutated PIK3CA in cancer, the majority of these studies were conducted in adenocarcinoma rather than in squamous cell carcinoma (SCC). Unlike those on adenocarcinoma, for which there have been numerous reviews, reviews on mutated PIK3CA in SCC are scarce and have seldom been published. Considering the literature gap, we composed this review paper with the intention of better understanding the clinicopathological significance of mutated PIK3CA in the context of SCC and better understanding how mutated PIK3CA influences the cellular behavior of SCC cells. In this review, we critically analyzed the current literature published regarding the occurrence of PIK3CA mutations in SCC (Fig. 1) to provide a new perspective for examining mutated PIK3CA in the context of SCC. The implications and ramifications of mutated PIK3CA in SCC have been highlighted in critical discussion, revealing the relationship between mutated PIK3CA and human papillomavirus (HPV) infection, malignant behavior of SCC cells in vitro, clinicopathological significance, survival, recurrence, response to chemotherapy and immune checkpoint inhibitors/blockers, radiotherapy, association with infiltration of immunocytes, association with expression of PD-L1 and so forth. It should be noted that those investigating the amplification of the PIK3CA gene in SCC or PIK3CA mutation in adenocarcinoma fall beyond the scope of this review and are not included in the discussion here.

## What squamous cell carcinoma (SCC) was

### Squamous cell carcinoma (SCC)

SCC is a malignancy originating from the squamous epithelium that normally covers organs, including the skin [1], mouth [2], esophagus [3], lung [4], cervix [5], and urinary tract [6]. On account of space limitations, it would be impracticable to list all of them here. Considering the tissue range where it arises, SCC is the most common cancer capable of metastasis [7]. With the same name, however, SCCs from different sites form a dramatically diverse group of cancers that present many differences in their symptoms, prognosis, and response to treatment. In terms of incidence, which is commonly observed in clinics, the majority of SCCs originate from the skin, head and neck, esophagus and lung. It has been well established that the risk of SCC is inextricably linked with environmental factors [8, 9], such as tobacco, alcohol, oral bacterial and viral exposure. SCC is strongly associated with a high risk of metastasis and morbidity and can result in significant mortality; thus, specialized surgical techniques for complete excision are needed [10]. Prior to metastasis to the lymph nodes, lung and other distant sites, the development of SCC is often uncontrolled, and SCC can invade nearby tissues or even metastasize to distant organs. Numerous studies have shown that the proliferation and metastasis of SCC cells are controlled by oncogenic signaling pathways, one of which is the PI3K-AKT-mTOR pathway [11, 12]. Several review papers on the PI3K-AKT-mTOR pathway in SCC have investigated this pathway from various perspectives, including intervention, targetability, and implications *for radioresistance* [11–16]. Here, we will no longer focus on this pathway. Instead, we refer readers to previously published review articles [11–16] that discussed the pathway at great length in the setting of SCC and who were interested in and wanted to learn more about this signaling pathway.

## Outline of the PI3K-AKT-mTOR pathway

Although numerous similar reviews have been published on this classical signaling pathway in different cancer settings [17–21], we feel that there is also a need to summarize the classical pathway here, which is considered a temporary brush-up. The phosphatidylinositol-3-kinase (PI3K) signaling pathway, which involves AKT and its downstream target mammalian target of rapamycin (mTOR) and is highly conserved throughout evolution from flies to mammals, has drawn intense attention from investigators as a pivotal regulator of cell proliferation and survival. The PI3K-AKT-mTOR pathway has been well established as an important pathway for cancer treatment. In the pathogenesis of SCC, oncogenic signaling, one of the hallmarks of cancer in the majority of solid tumors, is sustained through the activation of the PI3K-AKT-mTOR pathway. Activated PI3K phosphorylates and alters the cellular lipid membrane, leading to the localization of the protein kinase AKT to the membrane. Once activated, AKT initiates a cascade of protein phosphorylation that activates mTORC1, a key regulator of cellular growth and survival [22]. PI3K family members are classified into three types: class I, II and III. Among the three main subtypes of PI3K enzymes, class I enzymes are the most commonly dysregulated and most well studied in cancer. Class IA PI3K enzymes are heterodimers that include regulatory subunits (p85α, p55α, p50α, p85β, and p55γ) and one of the three 110 kiloDaltons (kDa) of catalytic kinases (p110α, p110β, and p110δ). Detailed illustrations concerning these subunits have been made repeatedly by previous reviews; we are not going to rehash them here but will refer readers who are interested in them for more information they want [17–21]. The critical roles of PI3K-Akt-mTOR signaling are foreshadowed by the discovery of mutations in genes encoding key components involved in the pathogenesis of human diseases, such as cancers. With next-generation sequencing, somatic alterations in genes encoding various members of the PI3K-Akt-mTOR signaling pathway have been frequently identified in cancers of various tissues of origin. PI3K-Akt-mTOR signaling can be constitutively activated as a result of these mutations. As we recalled the basic knowledge of the PIK-AKT-mTOR signaling pathway under normal physiological conditions above, we next discuss this pathway in the context of SCC we have concerned most.

### Aberrant PI3K-AKT-mTOR pathway activity in SCC

**Fig. 2:**
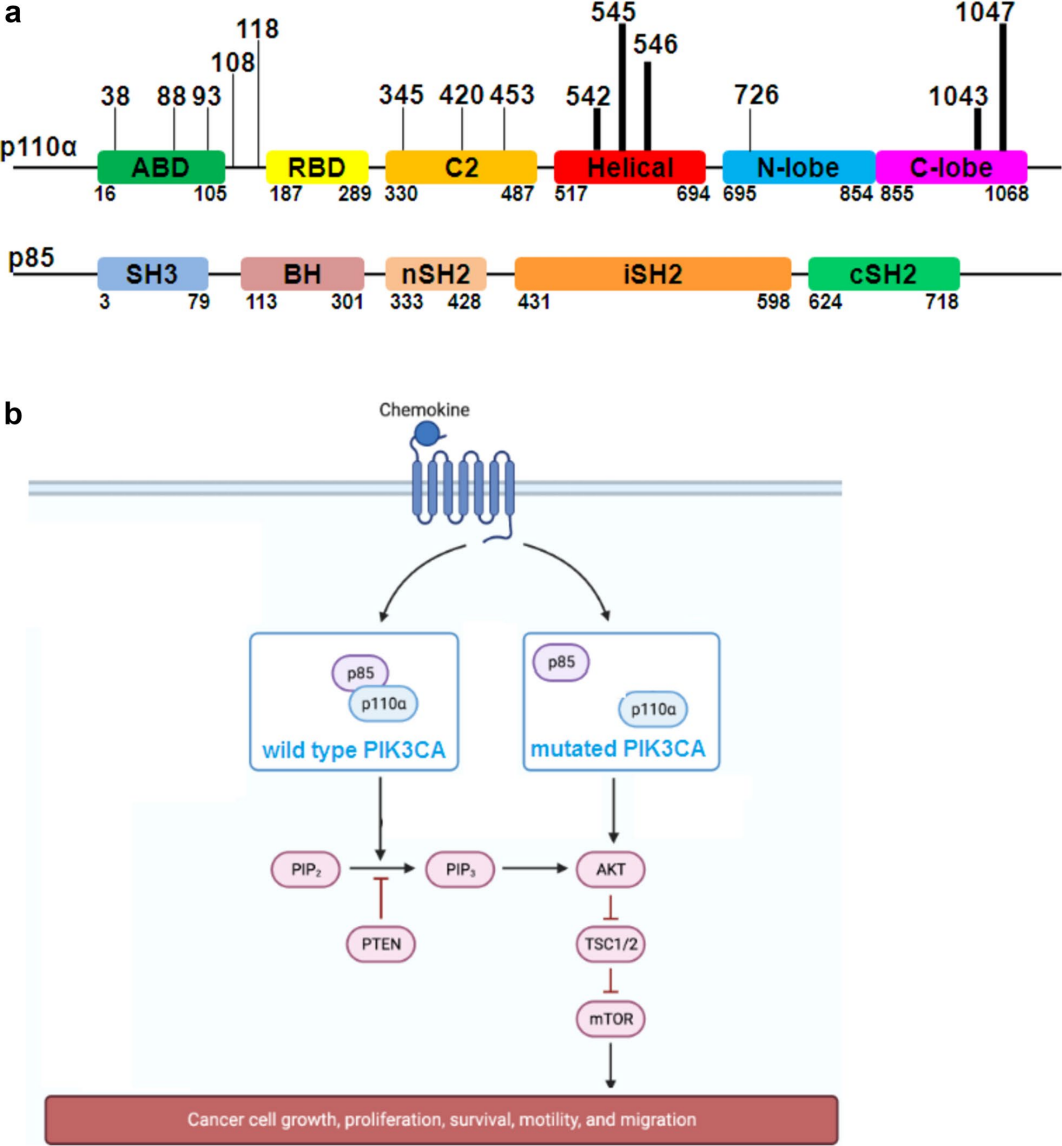
Schematic diagrams showing the mutational status of the PIK3CA gene and hyperactivating mechanism of the PI3K-AKT-mTOR signaling pathway. **a.** PIK3CA genetic structure and frequent oncogenic mutations identified in the TCGA database. **b.** Schematic illustrating the mechanism by which mutated PIK3CA hyperactivates the PI3K-AKT-mTOR pathway. The PI3K-AKT-mTOR pathway schema was credited to the drawing platform (https://www.biorender.com/)

Advances in DNA sequencing and analysis of human cancer genomes have revealed that the PI3K-Akt-mTOR pathway is commonly dysregulated in cancers arising from diverse tissues of origin. The ubiquity of PI3K-Akt-mTOR pathway activation has aroused much interest in the development of small molecule inhibitors targeting this pathway. As revealed by The Cancer Genome Atlas (TCGA) database analyses, mutations in the PI3K-mTOR pathway are dysregulated overall in a large number of cancers, and its importance for different cellular roles is critical in carcinogenesis, which justifies its use as an appealing target for drug invention [23]. According to in silico analyses of data from the TCGA database, numerous components of the PI3K-AKT-mTOR pathway were upregulated more frequently than any other pathway in cancer by mutation, loss, DNA methylation, amplification and translocation, with resultant activation of the pathway [13, 14]. Somatic mutations in key members of this signaling pathway, including PIK3CA, PIK3R1 and AKT [24], are known to be among the main causes of cancer. Among the gene mutations found to occur in several members of the PI3K-AKT-mTOR pathway in solid tumors, according to the literature, the mutation rate was highest in PIK3CA, accounting for 13%, followed by the mutation of phosphatase and tensin homolog (PTEN) (9%) [25]. Unlike wild-type PIK3CA, mutated PIK3CA can weaken the direct interaction between the PIK3CA and p85 subunits, which in turn hyperactivates the PI3K-AKT-mTOR pathway (Fig. 2). The original report investigating mutated PIK3CA led to hyperactivation of the PI3K-AKT-mTOR pathway in SCC derived from oral squamous cell carcinoma (OSCC) [26], wherein AKT was found to be highly phosphorylated in OSCC cell lines with PIK3CA mutations compared to that in their counterparts without mutations. This finding was subsequently replicated in a subsequent investigation of lung cancer [27] in which lung squamous cell carcinoma (LUSC) and lung adenocarcinoma (LAD) were confirmed. In contrast with that in LAD, PIK3CA mutation seems to be preferred for SCC, meaning that the mutation rate of PIK3CA was more frequently greater in LUSC than in LAD. Several studies support this conjecture. For instance, in lung cancer and esophageal cancer, a few studies [28, 29] revealed that mutations in PIK3CA were markedly more frequent in SCC than in adenocarcinoma. However, this suggestion has recently been challenged by a comparative study [30] performed between cervical squamous cell carcinoma (CESC) and cervical adenocarcinoma (CAC), which explicitly demonstrated that PIK3CA mutation rates did not differ significantly between CAC and CESC. Further research should be performed to investigate whether there are differences in terms of PIK3CA mutation rates between SCC and adenocarcinoma.

### Mutating PIK3CA could be a late molecular event in the pathogenesis of SCC

As stated earlier, although derived from different organs with similar pathological diagnostic morphologies, SCCs present many differences in their symptoms, prognosis and response to the same treatment regimen, which cannot be simply or sweepingly combined. Aside from these differences, SCC histopathologically evolves from normal epithelial to intraepithelial neoplasm to squamous cell carcinoma of different grades. Considering that this line of evolution eventually leads to SCC, one may wonder when PIK3CA is mutated. Currently, there are no definite answers available. Moreover, there has been little evidence exploring this question in the setting of SCC. Nevertheless, according to the limited evidence available, we can speculate that mutated PIK3CA is a late event in the pathogenesis of SCC. This conjecture was supported by Verlaat W et al.’s [31] investigation of cervical cancer, in which a somatic mutation in PIK3CA represented a late event during cervical carcinogenesis. Furthermore, more direct evidence from a study [32] comparing genomic alterations in the transition from cervical intraepithelial neoplasia (CIN) to microinvasive carcinoma (MIC) and cervical squamous cell carcinoma (CESC) revealed that PIK3CA was altered in the MIC and CESC either by mutation or amplification but not in the CIN, indicating that mutations such as PIK3CA, TP53, STK11 and MAPK1 are needed in the transition from CIN to MIC or CESC, in addition to human papilloma virus infection. Similarly, Kozak K et al. [26] reported that somatic mutations in the PIK3CA gene are likely to occur late in the development of OSCC. Nevertheless, Kashofer K et al. [33] detected the hotspot mutation of PIK3CA at E545K and E453K in the early stage of CESC with a microinvasive pT1 stage and reported that the mutation rates of PIK3CA E545K and E453K were 5/8 (62%) in CESC with pT1a, 7/15 (70%) in CESC with pT1b and 10/15 (66%) in CESC with pT2/3. These data indicate that PIK3CA mutation can occur early in the carcinogenesis of CESC. With the exception of these two separate studies [31, 32] mentioned above, mutations in the PIK3CA gene were significantly more frequent in smokers than in nonsmokers [27, 34], implying that there seems to be a relationship between PIK3CA mutations in SCC and environmental exposure, which could be an important molecular event in advanced SCC that needs to be substantiated with more compelling evidence. Fundamentally supporting this notion, Bumrungthai S et al. [35], analyzing the mutation of PIK3CA in CESC, found that PIK3CA mutation acquisition was positively correlated with the age of patients with CESC. That is, the older the patient is, the greater the mutation rate of PIK3CA. Information from the abovementioned studies indirectly suggested that PIK3CA mutation was likely a late molecular event in the carcinogenesis of SCC. More direct experimental evidence is needed to substantiate this conjecture.

### PIK3CA mutations in SCC

In addition to the mutation of TP53 [36], the PIK3CA mutation seems to be one of the most frequent genetic alterations in tumors despite differences in terms of incidence across tumors of different types. Although there is a lack of a clear consensus about the prevalence of mutated PIK3CA in cancers, a few recent studies with statistical analyses of the prevalence of PIK3CA mutations in cancers have shown that breast cancer has the highest mutation rate, followed by endometrial, bladder, colorectal carcinoma, and head and neck squamous cell carcinoma [37, 38]. It is well known that the majority of PIK3CA mutations involve either the kinase domain of PIK3CA, which leads to constitutive activation of the PIK3 signaling pathway, or the helical domain, resulting in a structural change lessening the intermolecular interplay between PIK3CA and the regulatory subunit p85 [39–41] (Fig. 2). Aside from mutations, an additional major abnormality of PIK3CA that affects the PI3K-AKT signaling pathway is gene amplification [42], which will be disregarded and not discussed here, as stated previously. Several seminal studies [43–46], using either whole-genome sequencing or whole-exome sequencing with large sample sizes, have repeatedly confirmed the heavy implications of hotspot PIK3CA mutations in esophageal squamous cell carcinoma (ESCC) clinical tissues. However, not all investigations regarding ESCC have revealed PIK3CA mutations. For instance, in a study examining mutated PIK3CA in Japanese patients with ESCC [47], in which only 44 patients were enrolled, the authors did not detect the mutation of PIK3CA. In another ESCC study enrolling 88 Japanese ESCC tissues, only two patients were found to have somatic mutations in exon 9 of PIK3CA, and no mutations were identified in exon 20 of PIK3CA. On the basis of these earlier reports, we can speculate with compelling evidence provided by Ko KP et al. [48] that compared with those of TP53, CDKN2A and NOTCH1, which are commonly altered in ESCC, the importance of PIK3CA mutations in the pathogenesis of ESCC is likely less important. We can also speculate that mutated PIK3CA alone could not be sufficient to induce ESCC, while the onset of ESCC could lead to the mutation of PIK3CA as a late molecular event.

## The genotype of mutated PIK3CA in SCC

### Hotspot mutations of PIK3CA in SCC

According to the frequency of PIK3CA mutations, the mutation sites in PIK3CA can be classified as frequent or infrequent. Approximately 80% of PIK3CA mutations in human cancers are observed in hot spot regions[49], that is, exon 9 (E542K [50] and E545K [50]) and exon 20 (H1047R [50]). In addition, the frequently reported mutations in PIK3CA include Q546K [28] and H1047L [51]. In contrast, the uncommon mutation sites reported on PIK3CA in SCC include M1043I [52], Y343C [53], R516K [54], S541S [54], N1068S [54], T1053A [54] and Y1021Y [54]. Other rare mutational sites that were tentatively unknown may be left to be further discovered. Next, we will pick up and expand on a few of them that frequently occur in the setting of SCC. Located in the PIK helical domain of PIK3CA, E542K is a hot spot mutation that leads to increased phosphorylation of Akt, independent of cell survival of growth factors and transformation in culture. Like E542K, E545K is another hotspot mutation located in the PIK helical domain of the PIK3CA protein. E545K increases the function of the PIK3CA protein, which can be demonstrated by increased phosphorylation of Akt in cell culture. Compared with the hotspot mutation sites E545K and E545G, which occur in the helical domain of exon 9 on PIK3CA, the H1047R and H1047Y mutations are located in the kinase domain of exon 20 on PIK3CA. Occurring in the PI3K/PI4K kinase domain of PIK3CA, the H1047Y mutation increased Akt phosphorylation, thereby activating downstream signals. Similarly, H1047R is another hotspot mutation of PIK3CA that is frequently detected in SCC. Similar to H1047Y, the H1047R mutation was also found in the PI3K/PI4K domain of the PIK3CA protein. To date, no study has compared the difference in the frequency of hotspot mutations between exons 9 and 20 of PIK3CA. However, compared with those in exon 9, the frequency of exon 20 PIK3CA mutations seems to show a slight trend toward being more common in ESCC [55]. This trend does not seem to occur in cutaneous squamous cell carcinoma (CSCC). For example, in another recent report that investigated the hotspot mutation of PIK3CA in 143 patients with CSCC [49], the frequencies of the E542K, E545K and H1047R PIK3CA mutations were 1.4% (2/143), 2.8% (4/143), and 0.7% (1/143), respectively. Retrieving the literature reporting various types of hotspot mutations in PIK3CA in SCC, it is not difficult to determine that the majority of these reports that met our inclusion criteria were published almost ten years ago. A few of them have been reported in the literature. Given the limited space, we tabulated these earlier reports in Table 1, which is presented below. It should be noted that when searching the related literature, some will be inadvertently overlooked or omitted, and we apologize for our failure to appreciate them in a timely manner.

**Table 1:** Hotspot mutations of PIK3CA in SCC

SCC types	Sample size	Mutation site	Mutation rate	References
CSCC	143 cases	E542K, E545K and H1047R PIK3CA	1.4% (2/143), 2.8% (4/143), and 0.7% (1/143) respectively	[49]
CESC	32 cases	E545K and E453K	5/8 (62%) pT1a, 7/15 (70%) pT1b and 10/15 (66%) pT2/3 CESC	[33]
VSCC	149 cases	E545K	8%	[56]
HNSCC	48 cases	E542K, T1025T and the five novel mutation unreported ever before	29%	[54]
CESC	166 cases	E545K	20%	[57]
LUSC	180 cases	E545K	5%	[58]
HNSCC	87 cases	E542K,E545K, H1047R	19.5%	[59]
ESCC	52 cases	A1634C	7.7%	[60]
pharyngeal squamous cell carcinoma	24 cases	E452K,E545K and H1047R	20.8%	[61]
HNSCC	54 cases	E542K, E545K and H1047R	29.4% (5/17)of HNSCC cell lines, 10.5% (2/19)of Indian No mutation(0/18)in Vietnamese	[62]
HNSCC	12 kinds of HNSCC cell lines	E542K	8.3%	[63]
HNSCC	33 cases	Hot spot in exon 9 and 20	0	[64]
OSCC	108 case tissue and 14 OSCC cell lines	E542K,E545K,E545G,Q546L,M1043V,H1047R,G1049S,G1049R	8/108 (7.4%) for tissues 3/14 (21.4%)for cell lines	[26]
nasopharyngeal squamous cell carcinoma (NPSCC)	46 cases	N521K,D549G	4.3%	[65]
OSCC	50 cases	E542K,E545K,A994A,T1025T	4%	[66]
Penile squamous cell carcinoma (PSCC)	30 cases	unspecified	10%	[67]

### Involvement in squamousness

Originally proposed by Schwaederle M et al. [68], the concept of “squamouness” actually referred to gene signatures with common molecular alterations, regardless of the organ of origin. The common molecular alterations include TP53, PIK3CA, CCND1, CDKN2A, SOX2, NOTCH 1, and FBXW7 aberrations but do not include KRAS alterations, which are significantly less frequent in SCC than in non-SCC histologies. In contrast, the aforementioned squamousness gene signature was significantly more frequent in SCC than in non-SCC histologies. The role of PIK3CA mutations in squamouness remains elusive. However, a seminal study by Garcia-Carracedo D et al*.* [69], who employed a genetically engineered mouse model, revealed that the PIK3CA mutation at H1047R strongly promoted the progression of OSCC induced by 4-nitroquinoline 1-oxide (4NQO) *in vivo*, explicitly indicating that the PIK3CA mutation at H1047R can exacerbate the progression toward OSCC. In support of this finding, Tan MT and associates [70], utilizing a transgenic mouse model harboring the PIK3CA mutation at H1047R, reported that the PIK3CA mutation accelerates the development of OSCC in a mouse model exposed to 4NQO. In light of the aforementioned literature [32], a comparison was made of the transition from CIN to MIC and CESC, showing that PIK3CA was altered in MIC and CESC either by mutation or amplification but not in CIN, explicitly indicating that driving mutations such as PIK3CA, TP53 and MAPK1 were needed in the transition from CIN to MIC/CESC in addition to human papilloma virus (HPV) infection.

### Association with HPV status

HPV infection, especially high-risk HPV infection, has been inextricably linked with the occurrence and development of SCC, irrespective of the different types involved. However, the relationship between HPV infection and PIK3CA mutation in SCC seems to be somewhat debated. For example, Feldman R et al [71] studied 735 patients with HNSCC and used a gene sequencing technique and showed that PIK3CA mutation was a frequent event, independent of HPV status. Studies with similar findings also included Wright AA et al.’s [30] comparative study on cervical adenocarcinoma and squamous cell carcinoma. In contrast, another study [59]involving a smaller sample size (81 patients) of patients with HNSCC revealed that PIK3CA mutation was significantly associated with HPV status. Specifically, compared with that in HPV-negative controls, the frequency of PIK3CA mutations was significantly greater in HPV-positive HNSCC patients. Similarly, Choschzick M *et al.* [72] reported that in vulvar squamous cell carcinoma (VSCC), in addition to NOTCH1 mutation, PIK3CA mutation (preferred at E542K and E545K) was also strongly linked to HPV infection. In contrast to VSCC, a recent study [73] involving a total of 171 patients with bladder squamous cell carcinoma (BSCC) did not reveal any striking difference in PIK3CA mutation between BSCC-positive or BSCC-negative patients with HPV. In view of these disparate findings, more research on this topic needs to be undertaken before determining the association between PIK3CA mutation and HPV status.

## The phenotype of mutated PIK3CA in SCC

### Promotion of malignant behavior

As stated above, somatic mutations have been identified in numerous genes, including PIK3CA, PTEN, Akt1 and other members of the PI3K-AKT-mTOR pathway, many of which have been clearly demonstrated to play important roles in promoting cancer cell proliferation and survival. Compared with wild-type PIK3CA, mutated PIK3CA was shown to promote the proliferation and migration of tumor cells. For example, Okudela K et al. [74], by using immortalized airway epithelium 16HBE14o- cells, showed that the effects of the PIK3CA mutation at K545E and H1047R were more remarkable than those of wild-type PIK3CA in the promotion of proliferation and invasion of lung squamous cell carcinoma (LUSC) cells. Similarly, Bonelli MA et al [75] reported that, regardless of the mutation at E545K or H1047R, mutated PIK3CA can accelerate the growth, migration and invasion of LUSC cells compared with those of control cells harboring wild-type PIK3CA. In addition, mutated PIK3CA promoted the epithelial mesenchymal transition process in LUSC cells. Similar to LUSC, in HNSCC [62], HNSCC cell lines harboring PIK3CA mutations displayed stronger colony formation efficiency and greater migration and invasive abilities than PIK3CA wild-type cell lines. Consistent with this research, another study in HNSCC performed by Kidacki M et al*.* [76] in which keratinocytes were used replicated the phenomenon that the PIK3CA mutation, regardless of the E545K or H1047R mutation site, significantly enhanced both migration and invasion relative to the wild type. Compared with hot spot mutation sites extensively reported to date, the mutation of PIK3CA at G1633A [77] has been comparatively underinvestigated. The mere evidence regarding the G1633A mutation in PIK3CA was that the G1633A mutation inhibited apoptosis in HNSCC cell lines treated with AG1478, an inhibitor of EGFR tyrosine kinase. In addition to this report, little is known about this mutation in SCC. To better understand the phenotype caused by mutated PIK3CA in the setting of SCC, we summarized the literature that is tabulated below (Table 2). As shown in Table 2, compared with the wild-type control, mutated PIK3CA promoted the growth, migration and invasion of SCC cells, irrespective of the mutation site or cancer type. However, little agreement has been reached on the sensitivity of SCC cells whose outcomes appear to be dependent on cancer type to inhibitors of the PI3K pathway caused by mutated PIK3CA.

**Table 2:** Phenotype of SCC cells in which PIK3CA was mutated

SCC cell lines	Mutation site	Phenotype	References
SiHa	E542K, E545K	Promotes the growth, sensitize cells to Alpelisib compared with wild type control	[78]
Caski, MS751	E542K, E545K	Up-regulates PD-L1, suppresses CD8 + T differentiation	[79]
primary cell cultures derived from CSCC	E545K	resistant to Gefitinib compared with control	[80]
Primary OSCC cell lines derived from genetically engineered mouse	H1047R	Sensitize to cisplatin and Alpelisib combination therapy compared with wild type	[69]
OSCC cell line CAL27	H1047R	less responsive to palbociclib compared to wild type	[81]
HNSCC cell line Cal-33	H1047R	Sensitize to Taselisib (GDC-0032)	[82]
LUSC cell line H596 and HCC2450	E545K,H1047R	increased growth, migration and invasiveness	[75]
normal oral keratinocytes(NOK)	E545K, H1047R	Promotes invasion via elevation of MMP1	[76]
immortalized airway epithelium 16HBE14o	K545E,H1047R	enhanced growth and migration	[74]
TSCC cell line CU-OP-2	E545K	Sensitize to Alpelisib	[83]

It should be noted that, in the scrutiny of these published papers, a serious weakness with these papers, however, is that most studies have only been carried out *in vitro* in SCC cell lines. *In vivo* animal models have seldom been used to complement *in vitro* experimental data. Given the distinctive differences between *in vitro* cell culture *and in vivo* physiological animal environments, more studies carried out with animal models in which PIK3CA was mutated at the hotspot are needed to resolve this controversy.

### Responses to cisplatin

What is going on when ESCC patients harboring PIK3CA mutations are exposed to first-line chemotherapy drugs, such as 5-fluorouracil or cisplatin? It is unclear. However, according to a recent report [84], there seems to be no relationship between them. For instance, by expanding the sample size, Zhang J and associates [84] studied 96 cases of ESCC tissues and constructed a PDX model and reported that PIK3CA gene mutation had no apparent impact on the growth of tumors affected by cisplatin or 5-fluorouracil in nude mice, indicating that there was no significant association between the presence of PIK3CA mutation and the likelihood of a response to cisplatin and 5-fluorouracil. Consistent with this report, exploiting a database of 104 patients with ESCC, Cui YP’s group [85] reported that patients with PIK3CA mutations benefited more from chemotherapy than those with wild-type PIK3CA. This study suffers from one hard fact that the authors did not explicitly define the drug names in regard to chemotherapy. In agreement with the conclusion reached by Cui YP’s group [85], Garcia-Carracedo D et al [69], who employed a genetically engineered mouse model of ESCC, revealed that the PIK3CA mutation at H1047R sensitized ESCC cells to cisplatin and BYL719, a specific inhibitor that potently inhibits the two most common PIK3CA somatic mutations at H1047R and E545K, strongly indicating that the PIK3CA mutation at H1047R enables OSCC cell lines to be sensitive to cisplatin. Similar findings have also been obtained from one deliberate investigation [78] conducted in cervical squamous cell carcinoma (CESC), where PIK3CA-mutant cervical cancer cells were more sensitive to the combination of BYL719 and cisplatin *in vivo*. In contrast, an *in vitro* investigation by Arjumand W et al [86] using CESC cell lines revealed that the PIK3CA-E545K mutation confers cisplatin resistance and a migratory phenotype in CESC cells. Although the findings reviewed above were mainly achieved *in vitro* in a cell culture system and lacked *in vivo evaluation*, combination therapy with cisplatin and a PI3K inhibitor may be worthy of consideration for SCC patients with PIK3CA mutations. Moreover, according to these reports, PIK3CA mutation has limited utility as a biomarker of the response to conventional treatment regimens prescribed for SCC, such as cisplatin.

### Responses to PI3K inhibitors

In contrast to reports on the response to chemotherapy drugs, such as 5-fluorouracil or cisplatin, there have been no conflicting reports available until now regarding the response of patients with PIK3CA mutations to PI3K inhibitors. A few studies have shown that tumor cells harboring PIK3CA mutations are more sensitive to inhibitors targeting the PI3K signaling pathway. For instance, in a study [87] involving both LUSC and lung adenocarcinoma tissues from lung cancer patients, PIK3CA mutations were found in 9% of LUSC patients and 0% of adenocarcinoma patients. Noticeably, tumor cells harboring PI3K mutations were shown to be highly sensitive to the PI3K inhibitor GDC-0941. In summary, in another study [88] on HNSCC in which a reverse-phase protein array technique was used, the authors revealed that cancer cell lines derived from HNSCC with PIK3CA mutations were sensitive to PI3K pathway inhibitors, whereas amplification status did not predict sensitivity. Similarly, PIK3CA mutations were found to predict the response to PI3K inhibitors in LUSC [89]. However, the notion that PIK3CA mutations are susceptible to PI3K inhibitors seems to have been challenged by a recent study conducted in cervical carcinoma [90] in which no striking association was observed between PIK3CA mutation and sensitivity to PI3K/AKT/mTOR inhibitors in cervical squamous cell carcinoma cell lines. Given these findings, further studies with a greater focus on evaluating the response of patients with mutated PIK3CA to PI3K inhibitors are warranted in SCC.

### Responding to targeted therapy

Unlike chemotherapy, targeted therapy complicates the management of SCC. By establishing a patient-derived xenograft (PDX) model in nude mice, Wu X et al. [91] demonstrated that ESCC tissues harboring PIK3CA mutations were insensitive to trastuzumab, a type of humanized monoclonal antibody used to treat breast cancer and gastric cancer, which has been infrequently used for ESCC. The significance of this study is that trastuzumab treatment is limited to patients with ESCC with PIK3CA mutations. In addition to trastuzumab, nearly identical findings were reported in HNSCC [92], in which hotspot mutations in PIK3CA and RAS can predict resistance to cetuximab, a monoclonal antibody targeting epidermal growth factor receptor (EGFR). A similar result was observed for the EGFR inhibitor. For instance, using a PDX mouse model, Bernat-Peguera A et al [80] revealed that cutaneous squamous cell carcinoma (CSCC) tumors harboring the PIK3CA-E545K mutation were resistant to gefitinib, an inhibitor of EGFR. In contrast to clinical results using trastuzumab, another active investigation was conducted in CESC [93], demonstrating that pembrolizumab monotherapy induced complete remission in a recurrent CESC patient with the PIK3CA-E545K mutation, implying that the PIK3CA-E545K mutation could be a potential marker for pembrolizumab treatment in CESC. These data undoubtedly showed that different types of SCC carrying the same PIK3CA-E545K mutation when treated with different monoantibodies could lead to different clinical outcomes, implying the limited value of mutated PIK3CA as a response marker to targeted therapeutic agents.

### Responding to radiotherapy

In addition to chemotherapy, radiotherapy is another chief means of treatment that has been widely applied in the standard therapy for SCC. Nevertheless, radioresistance (RR) has been identified as an important cause of treatment failure following chemotherapy, but its underlying mechanism remains largely unknown despite the large amount of available literature. To date, no relationship has been established between PIK3CA mutation and RR in SCC. However, a recent article [94] enrolling one hundred forty-three patients with HNSCC included in the molecular screening of the ProfiLER database suggested that PIK3CA mutation plays a significant potential but unclear role in RR. A similar indication can also be found in another study regarding CSCC contributed by the same authors [95], who, using the ProfiLER trial platform, analyzed the PIK3CA mutation in CSCC, revealing that the PIK3CA mutation was most common among all mutated genes in patients with CSCC who initially underwent radiotherapy. Although there is a lack of an explicit causal association between PIK3CA mutation and RR from these two serial investigations performed in the context of SCC, it would be no hard to see that PIK3CA mutation was heavily implicated in the RR of SCC. Consequently,

However, further studies that consider the relationship between PIK3CA mutation and the RR in SCC patients need to be performed.

## Clinicopathological significance of mutated PIK3CA

Given that the majority of reports concerning mutated PIK3CA were conducted in adenocarcinoma, the clinicopathological meaning of PIK3CA mutations has rarely been documented in SCC, especially in cases with limited sample sizes. For example, Kim HS et al. [51] sequenced 388 ESCC samples and detected PIK3CA mutations, revealing that the mutation rate of PIK3CA was 1.5% (6/388). Specifically, 5 patients had the E545K mutation in exon 9, while one had the H1047L mutation in exon 20. Unfortunately, these authors only analyzed the clinicopathological meaning of PIK3CA amplification, which was found to be associated with tumor stage and grade in this investigation. They did not provide any information regarding the significance of PIK3CA mutations. This may be partly due to the limited number of patients after stratification by PIK3CA mutation status. Using RNA sequencing (RNA-seq) data from The Cancer Genome Atlas (TCGA) database, we identified a total of 81 patients with ESCC. Among these 81 patients, 11 had PIK3CA mutations, and the remaining 70 had wild-type PIK3CA. After detailed statistical analyses, mutated PIK3CA was found to be unassociated with T, N and M classifications and clinical stage (unpublished). The mutation rate of PIK3CA appears to be greater in oral squamous cell carcinoma (OSCC) than in ESCC. In a study [26] of 108 OSCC patients, a striking correlation was found between PIK3CA mutation and clinical stage. Specifically, the frequency of PIK3CA mutations was markedly greater in the IV stage than in the I-III stage. Considering the low rate of PIK3CA mutation, additional studies with larger sample sizes are warranted to understand the clinicopathological meaning of PIK3CA mutations in SCC.

### Prognostic significance of mutated PIK3CA

The prognostic relevance of mutational PIK3CA in SCC has been controversial, and no general agreement has yet been reached. ESCC is no exception. The first report that PIK3CA mutation was closely related to a longer prognosis in ESCC was provided by Shigaki H et al. [55], who, using 219 Japanese ESCC samples by means of pyrosequencing, showed that Japanese patients with PIK3CA mutations in exons 9 and/or 20, regardless of whether single or multiple mutations occurred concomitantly, had significantly better disease-free survival and overall survival. In addition, the authors also evaluated the mutation frequencies of PIK3CA, which is commonly detected in clinical samples. The H1047R mutation rate was highest (35/219), followed by that of E545K (16/219), E542G (8/219), E545G (5/219), and E1047Y (4/219). In contrast, another study of 406 Chinese ESCC patients revealed that PIK3CA mutations were not associated with overall survival. However, for patients with a family history of cancer or patients aged younger than 59 years, PIK3CA mutation was shown to be significantly associated with a shorter overall prognosis. Consistent with this observation, Lee H et al [96] studied 64 patients with ESCC and revealed that PIK3CA mutation was strongly linked to poor prognosis. Similar findings can also be seen when referencing the separate studies concerning SCC by Munari FF et al. [97] and Beaty BT et al. [98], where PIK3CA mutations were argued to be significantly associated with lower survival when compared to wild-type patients. Aside from PIK3CA mutation itself, there has been a suggestion [99] that mutated PIK3CA, together with other mutations that are frequently observed, such as TP53, HRAS or CDKN2A, seems to be linked to more inferior outcomes in patients with vulvar squamous cell carcinoma (VSCC). Nevertheless, consistent with the conclusion drawn by Shigaki H et al. [55], in another study involving in silico analyses of 104 Chinese patients who underwent next-generation sequencing [85], the authors showed that PIK3CA mutation was associated with longer overall survival in patients with ESCC. Similar conclusions can also be drawn in studies by Yokota T et al [100], Liu SY et al [101] and McGowan M et al [102], in which PIK3CA mutation was shown to be strongly associated with better prognosis. These conflicting results were difficult to interpret and compromise, and the reasons leading to the discrepancy between these studies were unknown, which could be technical and may be due to the different clinical samples, different sample sizes and different detection methods leading to different detection sensitivities. On the other hand, the prognostic value of mutated PIK3CA in different types of SCC is difficult to determine.

### Association with relapse

Previous studies on PIK3CA expression in SCC reported that PIK3CA expression was strongly associated with the recurrence of SCC [103, 104]. However, there has been little agreement on the association between mutated PIK3CA and the recurrence of SCC.

In LUSC, McGowan M *et al.* [102], using 308 cases of LUSC, showed that the PIK3CA mutation was most frequently in exon 20, and the mutation rate was 11.4% (35/308). Importantly, patients with PIK3CA mutations were shown to be less likely to experience recurrence than nonmutated patients. Consistent with this observation, in another study enrolling 210 patients with ESCC, Liu SY et al*.* [101] revealed that the proportion of patients with PIK3CA mutations in exon 9 was 22.9% (48/210). Survival analyses revealed that patients with PIK3CA mutations in exon 9 were less prone to recurrence than patients with wild-type PIK3CA. In line with these findings, a similar report was conducted in ESCC by Song B *et al.* [85], in which data from 104 patients with ESCC were collected; the authors found that ESCC patients with mutations in PIK3CA had longer survival times than those without PIK3CA mutations. A synthesis of these studies appears to suggest that ESCC patients with PIK3CA mutations are less likely to experience recurrence. In contrast to these reports, which are basically in line with each other, there is conflicting evidence showing the opposite. For example, in ESCC, Wang L *et al.* [103], employing 406 patients with ESCC, discovered that thirty somatic point mutations (30/406, 7.4%) were identified in exon 9, whereas no mutations were detected in exon 20. Statistical analyses did not support that PIK3CA mutations were associated with local recurrence. In addition to ESCC, another study performed in oropharyngeal squamous cell carcinoma (OPSCC), Beaty BT and associates [98], revealed that patients with PIK3CA mutations easily experienced recurrence compared with those without mutations in PIK3CA. However, whether mutated PIK3CA can be used as a predictor for the recurrence of SCC remains controversial.

**Fig. 3:**
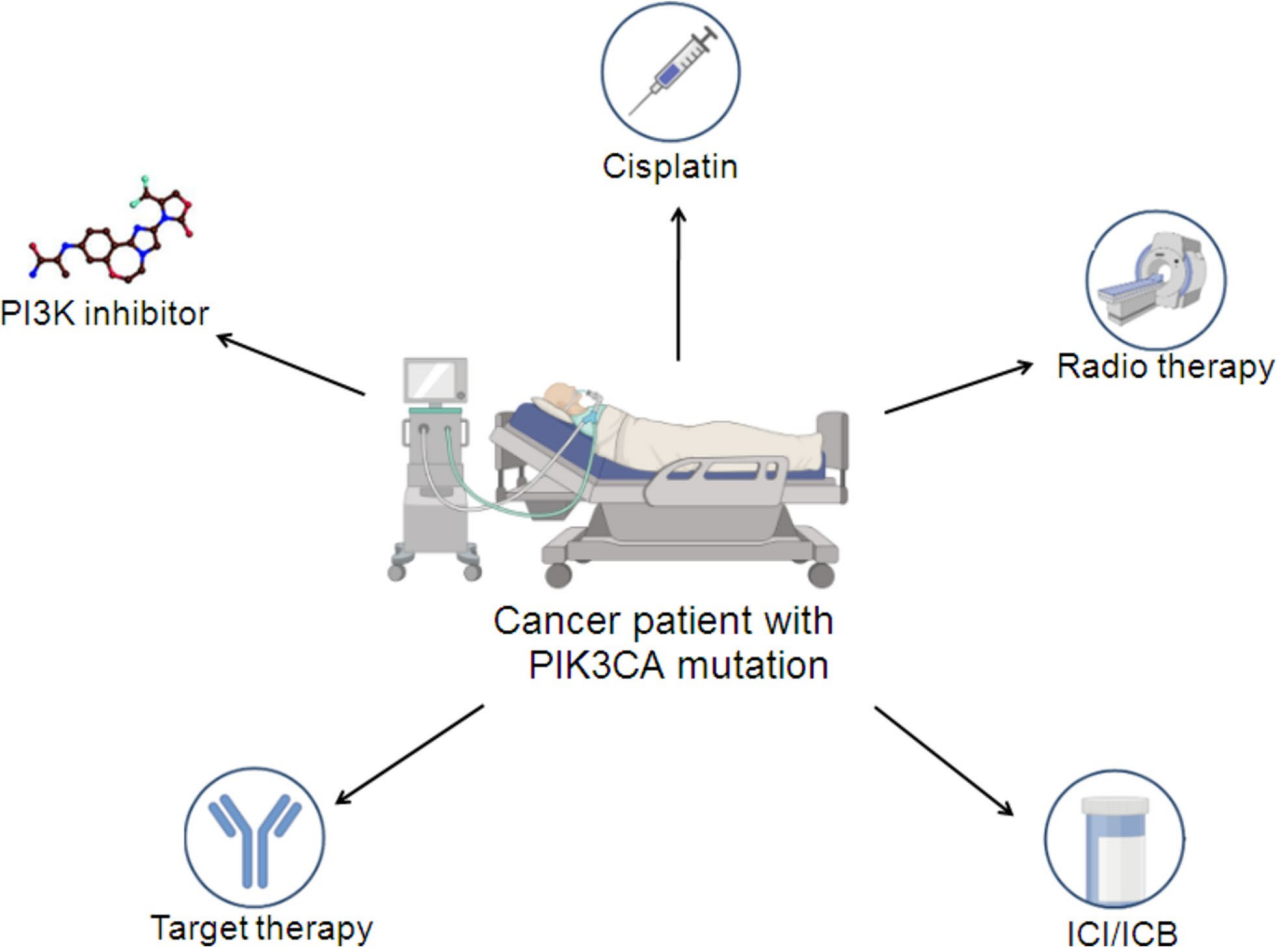
Schema depicting the response of patients with SCC with mutated PIK3CA to different therapeutic regimens. The icons denoting cisplatin, PI3K inhibitor, target therapy, radiotherapy and immune checkpoint inhibitors or blockers (ICIs/ICBs) were credited to the free-trial version of the online drawing platform (https://www.biorender.com/)

Currently, the literature closely linked with mutated PIK3CA in SCC shows that mutated PIK3CA is oncogenic and exacerbates the malignant behaviors of SCC cells *in vitro*. Although the clinicopathological, prognostic and recurrent relevance of mutated PIK3CA in SCC tissue are controversial, this discrepancy highlights the complex role of mutated PIK3CA, which may be tissue dependent. In the following sections, we will focus on the response of SCC cells with mutated PIK3CA to different therapeutic regimens, including conventional cisplatin, PI3K inhibitors, targeted therapy, immune checkpoint inhibitors/blockers (ICIs/ICBs) and radiotherapy (Fig. 3).

## Acquired resistance to immune checkpoint inhibitors (ICIs)

Compared to traditional chemoradiotherapy, immune checkpoint inhibitors (ICIs, some references that use the term blockers or ICBs) have become increasingly important in SCC therapy because the immunosuppressive tumor microenvironment plays an essential role in the treatment of SCC. However, the connection between PIK3CA mutation and resistance to ICIs in SCC remains obscure. However, analyses from the scarce literature available implicitly suggest that SCC cells with mutated PIK3CA seem to have acquired resistance to ICIs. As a good example supporting this, in a deliberate investigation [105] concerning acquired resistance to ICIs, the authors compared the molecular variation in pre- and post-ICI paired tumor samples derived from HNSCC patients with acquired resistance, employing a variety of approaches, including whole-exome sequencing, RNA sequencing and multiplex immunohistochemistry, and revealed that resistant HNSCC tissue harbored an E542K mutation in PIK3CA, which preliminarily seemed to reveal the relationship between the PIK3CA-E542K mutation and acquired resistance to ICIs. Given the pretty limited sample size the authors employed, the relationship needs to be robust using larger sample size. In contrast, after detailed statistical analyses, another study [106] enrolling the clinical data of HNSCC patients collected by the authors and TCGA database showed that PIK3CA mutations (HR = 0.45, *p* = 0.021) were a potential predictor of ICI efficacy and that PIK3CA mutations correlated with good overall survival in HNSCC patients, suggesting that PIK3CA mutations could identify patients who would benefit from ICI therapies in HNSCC patients. Consistent with these findings, a similar conclusion has been drawn for cervical squamous cell carcinoma (CECC) [107], revealing that patients with PIK3CA-E545K presented only a partial response to treatment with PD-1 inhibitors. According to the above conflicting findings, the causal relationship between PIK3CA mutation and acquired resistance to ICIs in SCC remains to be established. Whether PIK3CA mutagenesis can lead to acquired resistance to ICIs is largely unknown and warrants further experimental confirmation.

### Association with infiltration of immunocytes

However, investigations of the association between PIK3CA mutation and the infiltration of immunocytes in SCC are limited. Analyses from the literature showed that the related studies were either conducted in silico by resorting to different algorithms or through single-cell sequencing techniques. Undoubtedly, the association between PIK3CA mutation and the infiltration of immunocytes in SCC could vary depending on the different algorithms adopted by the authors. Therefore, care should be taken when interpreting such results. A case in point is that in sophisticated in silico analyses of HNSCC [108], where an immune-related gene prognostic index (IRGPI) was constructed to better molecularly subtype patients with HNSCC to benefit from therapy with immune checkpoint inhibitors (ICIs), the PIK3CA mutation rate was found to be high, along with high infiltration of B cells and M0 and M2 macrophages in the subgroup with a low IRGPI, which benefited less from ICI therapy than did the subgroup with a high IRGPI. However, the authors did not analyze the correlation between PIK3CA mutation and the infiltration intensity of B cells or M0 or M2 macrophages. Moreover, the authors did not relate the specific mutation site that occurred to PIK3CA in HNSCC of their own. A similar trend in which PIK3CA mutation seems to be closely associated with B-cell infiltration was largely reproduced in another recent study regarding ESCC [109], in which ESCC was classified into four different subtypes. One subtype is the immune suppression subtype, in which high infiltration of B cells and NK CD56^bright^ cells is associated with a significantly greater tumor mutational burden (TMB) than the immune modulation subtype. TMB included PIK3CA mutations. Importantly, patients with ESCC of the immune suppression subtype were less likely to benefit from ICI therapy. Although no explicit notion has been formulated that a high PIK3CA mutation is related to the infiltration of B cells and is poorly responsive to ICIs in SCC according to the two studies reviewed above [108, 109], reasonable inference leads us to hypothesize that SCC patients with a high PIK3CA mutation burden are unlikely to benefit from ICI therapy. In line with this hypothesis we proposed here, a supporting observation has been reported for cervical squamous cell carcinoma (CECC) [107], showing that patients with PIK3CA-E545K mutations showed a partial response to treatment with PD-1 inhibitors. Considering these findings, further experimental investigations are needed to determine the relationship between mutated PIK3CA and the infiltration of immunocytes in SCC.

### Mutated PIK3CA upregulates PD-L1 expression

In cancer immunotherapy, the PI3K/AKT/mTOR signaling pathway is closely linked to the expression of PD-L1. Inhibitors of this pathway could consequently downregulate the expression of PD-L1 in cancer [110, 111]. Nonetheless, previous research findings regarding the linkage between mutational PIK3CA and PD-L1 expression have been inconsistent and even somewhat contradictory [102, 112, 113]. These controversial observations were primarily made in patients with adenocarcinoma. In contrast to that in adenocarcinoma, it has been consistently reported that there is no association between PIK3CA mutation and PD-L1 expression in SCC, regardless of the specific cancer type [96, 102, 114, 115]. By analyzing these studies, we can see that these investigations were almost all correlational analyses, without any experimental evidence from an *in vitro* cell culture system. In contrast to these earlier findings, a recent study [93] performed in CESCs revealed that the PIK3CA-E545K mutation leads to the striking upregulation of PD-L1 expression, both at the mRNA and protein levels. Moreover, the differentiation of CD8+ T cells was also suppressed in the presence of the PIK3CA-E545K mutation. In addition, utilizing a cell culture system, the authors demonstrated [93] that PIK3CA mutation was able to increase the mRNA and protein levels of PD-L1 in CSCC cell lines. It remains to be determined whether there is a relationship between PIK3CA mutations and the expression of immune checkpoint proteins other than PD-L1 in SCC. In light of scarce evidence, considerably more work investigating the mechanism by which mutated PIK3CA regulates PD-L1 will need to be done in the future.

### PIK3CA mutation, not alone

In various malignancies other than SCC, PIK3CA mutations do not occur alone and more often than not coexist with other oncogenic mutations, such as TP53 and EGFR in SCC [116, 117]. This concomitant association between PIK3CA mutations and other oncogenic mutations, such as RAS or EGFR mutations, has important implications for the treatment of cancer. For patients with simultaneous PIK3CA and RAS or BRAF mutations, inhibitors targeting the PI3K-AKT-mTOR pathway are insufficient to achieve a remarkable antitumor effect because RAS or BRAF mutations are common in patients with PIK3CA mutations. It would therefore be necessary to determine the mutational status of RAS and BRAF in addition to the PIK3CA mutation. Some experiments [91, 92] have suggested that when facing multiple oncogenic mutations in SCC, the combination of signaling pathway inhibitors with chemotherapy drugs would be better than the use of inhibitors or chemotherapy drugs alone in terms of suppressing tumor efficiency. However, these observations need to be confirmed in additional experiments, especially using *in vivo* mouse models.

## Summary and perspective

In the pathogenesis of SCC, PIK3CA mutation is more likely a late event that is often associated with advanced-stage cancer. According to the literature, mutations in PIK3CA can enhance malignant behaviors and insensitivity to chemotherapy drugs in SCC cells. On the other hand, mutated PIK3CA is sometimes not necessarily detrimental, as anticipated. When treating SCC, the combined use of PIK3CA inhibitors could confer benefits to patients by overcoming or decreasing the acquired resistance of SCC cells to chemotherapeutic drugs. In summary, we propose that the mutational status of PIK3CA should be considered. With the deepening understanding of PIK3CA mutations in the tumor microenvironment of SCC, more biochemical functions of mutated PIK3CA will be uncovered and delineated in the future.

## Data Availability

Data availability is not applicable to this article, as no new data were created in this study.
